# Modified Tracheal Traction Exercise Reduces the Incidence of Dysphagia in Patients with Multilevel Anterior Cervical Discectomy and Fusion

**DOI:** 10.1111/os.14166

**Published:** 2024-07-18

**Authors:** Jiasen Wei, Fudong Li, Jingchuan Sun, Zhenjun Zhu, Rui Shi, Jiangang Shi, Kaiqiang Sun

**Affiliations:** ^1^ Department of Spinal Surgery Xinxiang Central Hospital Xinxiang China; ^2^ Department of Spinal Surgery Shanghai Changzheng Hospital, Naval Medical University Shanghai China; ^3^ Department of Spinal Surgery The Fourth Clinical College of Xinxiang Medical University Xinxiang China

**Keywords:** anterior cervical discectomy and fusion, complications, dysphagia, modified tracheal traction exercise, multilevel fusion

## Abstract

**Objectives:**

Dysphagia, an impairment in swallowing, is a frequent and debilitating complication for patients undergoing anterior cervical discectomy and fusion (ACDF), a common surgical treatment for degenerative cervical myelopathy (DCM). This retrospective study aimed to assess the efficacy of modified tracheal traction exercise (MTTE) in alleviating postoperative dysphagia and improving clinical outcomes for these patients.

**Methods:**

A cohort of 143 patients underwent multilevel fusions, equally distributed between MTTE (*n* = 75) and traditional tracheal traction exercise (TTTE) (*n* = 68) groups. Demographic parity was observed in gender distribution, age averages (MTTE: 51.43 ± 11.25 years; TTTE: 52.35 ± 10.43 years), body mass index (BMI), comorbidities, fusion segments, and preoperative hospitalization days. Surgical duration differences were assessed. Clinical outcomes, dysphagia incidence, blood loss, postoperative complications, Cervical Japanese Orthopedic Association (c‐JOA) scores, and functional outcome swallowing scale evaluations were conducted. Univariate and multivariate logistic regression analyses were used to explore factors influencing dysphagia.

**Results:**

Modified tracheal traction exercise demonstrated advantages with a significantly lower dysphagia incidence (25.33% vs. 44.12%, *p* = 0.018), reduced blood loss (102.03 ± 17.04 vs. 113.46 ± 14.92, *p* < 0.001), shorter surgical durations (159.04 ± 9.82 vs. 164.41 ± 12.22 min, *p* = 0.004), and fewer postoperative complications (choking cough, cerebrospinal fluid leakage, and hoarseness). Postoperative c‐JOA scores at 2 and 6 weeks favored MTTE, but no significant differences were observed at 12 and 24 weeks. Functional outcome swallowing scale evaluations favored MTTE with significantly higher percentages of “normal” and lower incidences of “mild” and “moderate dysphagia” at various postoperative intervals compared to TTTE. Factors significantly associated with dysphagia included MTTE, age, and BMI according to logistic regression analyses.

**Conclusion:**

Modified tracheal traction exercise demonstrates superior short‐term outcomes in multilevel ACDF, showcasing reduced dysphagia incidence, blood loss, and specific postoperative complications. Notably, factors contributing to dysphagia, including operation technique and patient‐related variables, emphasize the significance of MTTE and patient characteristics in optimizing postoperative outcomes in multilevel ACDF procedures.

## Graphical abstract

The procedure of modified tracheal traction exercise (MTTE). The patient is in supine position and a U‐shaped neck cushion is used to achieve cervical hyperextension. The operator confirms the location of thyroid cartilage, and then the thyroid cartilage is pushed to the left side at least 1 cm across the midline of the anterior neck. Before returning to the starting position, this status should be held for 5 s, swallowing when the pushing is performed.

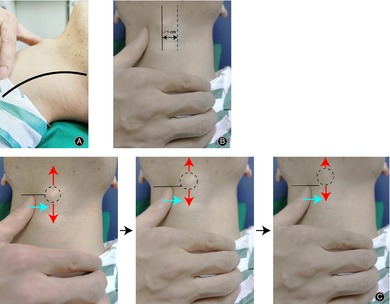

## Introduction

Degenerative cervical spondylosis is a common disease worldwide, which often causes severe injuries and compression to the spinal cord and nerve roots. Conservative treatment is frequently ineffective for patients with multilevel and severe degenerative cervical myelopathy (DCM), and timely surgical treatment is often recommended. Anterior cervical decompression and fusion (ACDF) has been widely used in clinical treatment of DCM. However, the complications related to ACDF cannot be ignored.^1^, ^2^, ^3^ Dysphagia after anterior cervical surgery (DAACS) is a frequent complaint of anterior cervical spine surgery.^4^, ^5^ Dysphagia is defined as an impairment in swallowing function during eating and drinking,^6^ which seriously affects the quality of life of patients and markedly increases the economic burden.

The occurrence rate of DAACS reported in papers ranged from 2% to 71%.^7^, ^8^ Previous research suggested that many factors may be associated with DAACS, although the exact cause is not well understood. Liu *et al*. reported that several factors were known as predisposing factors for DAACS, which include inadequate preoperative preparation, plate fixation, sex, multiple‐level fusion, location of surgical levels, the use of implants, and longer operative time.^9^ Several studies have reported that various strategies could be effective treatments or prevention approaches for postoperative dysphagia, such as dynamic retraction in the process of surgery, perioperative steroids, monitoring the endotracheal tube cuff pressure, using low‐profile plates, and tracheal traction exercise (TTE).^10^, ^11^, ^12^, ^13^, ^14^ It was reported that dynamic retraction in the surgical procedure can decrease the incidence of dysphagia.^11^ Curto *et al*. reported that local steroids significantly reduced the incidence and severity of dysphagia after cervical disk replacement surgery.^12^ The Zero‐P implant system was also reported to be relevant to the decreased incidence of dysphagia.^14^ The results of a randomized controlled trial revealed that a 20‐mmHg endotracheal tube cuff pressure could be associated with the lower incidence of dysphagia.^13^

Traditional tracheal traction exercise (TTTE) treatment has been commonly used for patients who will receive anterior cervical surgery. Although a previous study investigated the role of TTTE in alleviating DAACS and most patients pretreated with TTTE were in good condition, some patients who accepted multilevel fusion still showed DAACS.^10^ Therefore, we supposed that additional factors for DAACS, such as the lack of adaptative ability to the postoperative state, need to be considered. It was reported that multilevel ACDF can significantly increase the cervical lordosis, which has been proved to be associated with dysphagia because patients are not adaptive to the state of excessive lordosis in the cervical spine that exceeds the natural lordotic curvature.^15^ Further, the overuse of metal retractors in the tracheoesophageal groove has been reported to be a risk factor of dysphagia,^16^ which might not only cause damage to the esophagus but also increase the difficulty of adapting to the postoperative state. Chewing sugar‐free gum has been shown to stimulate saliva production, which can help in the early recovery of bowel function and improve swallowing reflexes after surgery.^17^ This practice can enhance postoperative recovery by promoting better oral hygiene and stimulating digestive functions, which indirectly supports swallowing function. In addition, the use of a U‐shaped neck cushion helps in achieving cervical hyperextension, which can optimize the surgical field and reduce tension on the trachea and esophagus during surgery.^18^ This positioning aid has been associated with improved postoperative outcomes, including reduced dysphagia, by minimizing tissue stress and promoting better anatomical alignment.^18^ Further, orofacial exercises, such as alternating between blowing, laughing, and grinning, might strengthen the muscles involved in swallowing and improve overall orofacial function, which might enhance neuromuscular coordination and facilitate better recovery of swallowing functions after surgery. The above beneficial strategies were included in the modified tracheal traction exercise (MTTE). Despite various strategies aimed at improving postoperative dysphagia, its incidence remains significantly unmitigated. Traditional measures such as TTTE, dynamic retraction, local steroid injections, and low‐profile plate use have not comprehensively resolved the issue. Therefore, novel and effective strategies are required.

The primary research purpose of this study is twofold: first, to compare the effects of MTTE and TTTE on postoperative swallowing difficulties in patients undergoing multilevel anterior cervical discectomy and fusion (ACDF), and second, to verify the effectiveness of MTTE in alleviating postoperative dysphagia and improving clinical outcomes.

## Methods

### Patients

To initially explore the prevention effects of MTTE on postoperative dysphagia, we conducted a retrospective study including patients received multilevel ACDF for the treatment of cervical radiculopathy or cervical myelopathy from January 2018 to December 2022 in our institution. This study was approved by the Ethics Committee of Shanghai Changzheng Hospital (No.2022SL037). All patients provided informed consent to use their clinical information for scientific research.

The inclusion criteria are (i) patients diagnosed with cervical radiculopathy or cervical myelopathy; (ii) patients who had accepted multilevel ACDF (≥3 levels); and (iii) patients with complete medical records. The exclusion criteria for this study are as follows: (i) gastro esophageal reflux; (ii) esophageal cancer; (iii) cervical operations performed before this surgery; (iv) dysphonia, hoarseness, or choking cough when drinking water before surgery; and (v) osteophytes, bony spurs on the margin of the vertebral body, were observed on the preoperative X‐ray film. To examine whether MTTE could reduce the rate of DAACS, the patients were divided into an MTTE group (patients received MTTE) and a TTTE group (patients received TTTE) according to the procedure they received before surgery.

The sample size was calculated using the following formula:
(1)

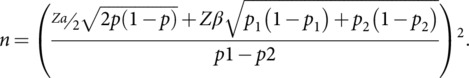




Based on previous studies and clinical relevance, we assume a reduction in dysphagia incidence from 40% in the TTTE group and around 16%–22% in the MTTE group. The significance level (α) was set as 0.05 and the power (1−β) was set as 0.80. The result of sample size calculation demonstrated that each group (MTTE and TTTE) would need approximately 62 participants to detect a statistically significant difference with 80% power and a 5% significance level.

### Modified Tracheal Traction Exercise

**FIGURE 1:**
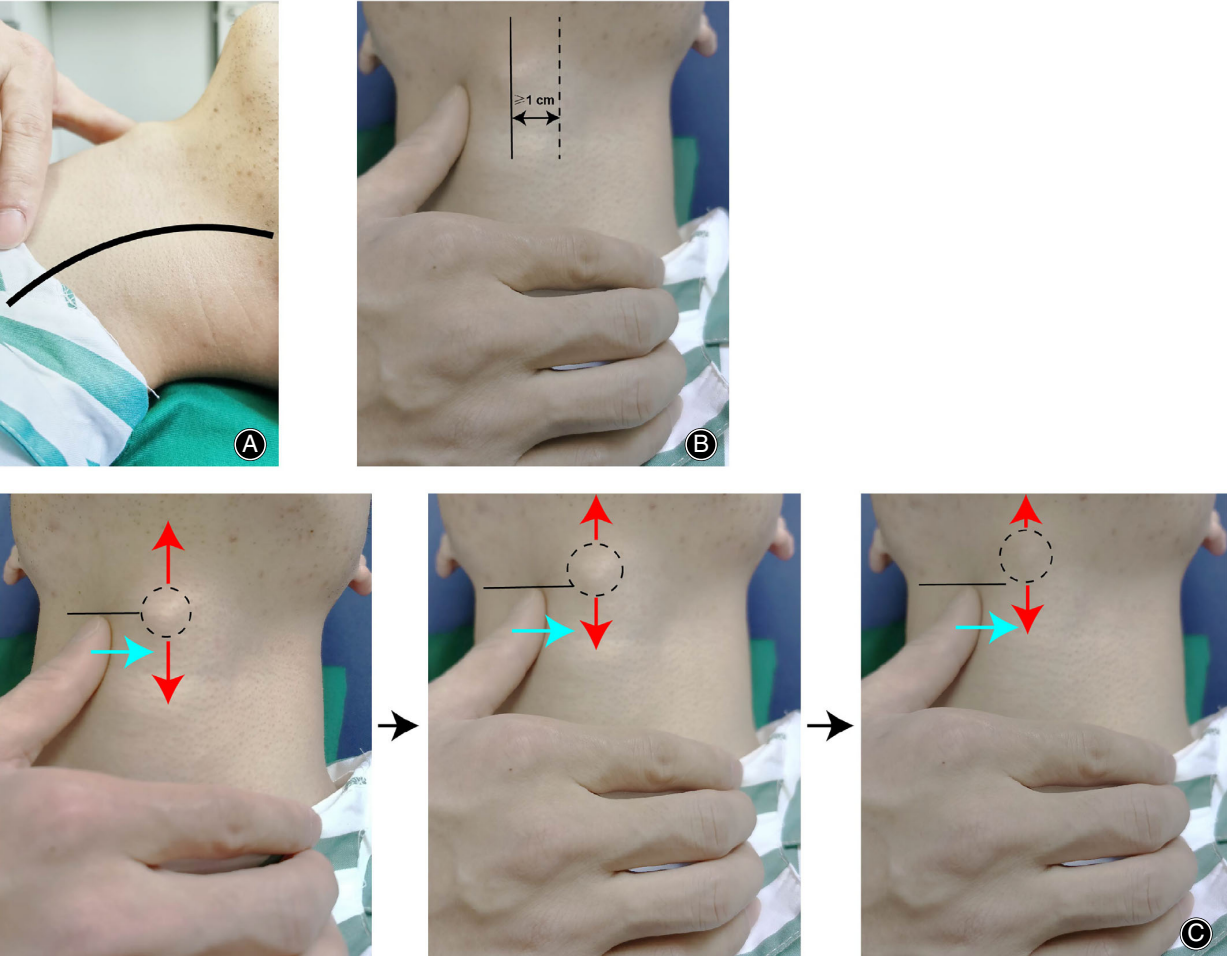
The procedure of modified tracheal traction exercise (MTTE). (A) The patient is in supine position and a U‐shaped neck cushion is used to achieve cervical hyperextension. (B) The operator confirms the location of thyroid cartilage, and then the thyroid cartilage is pushed to the left side at least 1 cm across the midline of the anterior neck and, before returning to the starting position, this status should be held for 5 s. (C) Swallowing when the pushing is performed.

To increase patients’ adaptability to the postoperative state, we introduced the MTTE technique, which has been improved based on TTTE. The TTTE procedure is as follows: patients lie in the supine position, with a pillow under shoulders; the operator stands on the right side of the patient, pushing the tracheal leftward at least 1 cm across the midline of the anterior neck. MTTE is carried out as follows:

1. Patients were placed in supine position and a U‐shaped neck cushion was used to achieve cervical hyperextension (Figure 1A).

2. The operator sat on the patient's right side and confirmed the location of thyroid cartilage (Figure 1B); the thyroid cartilage was pushed gradually to the left side at least 1 cm across the midline of the anterior neck and, before returning to the starting position, this position was held for 10 s (Figure 1B).

3. Patients were required to swallow when the pushing was performed (Figure 1C). The above procedures were performed 0.5 h postprandial three times a day, lasting 30 min each time until the day before surgery.

4. Patients began chewing sugar‐free gum for functional training 3 days before surgery, and the time was performed after each completion of tracheal movement training, with two pills per time for 15 min each time. When the vital signs were stable and the patients were conscious after surgery, they chewed sugar‐free chewing gum for 15 min each time after meals, two pills each time, until the 7th day after surgery.

5. Patients lie on the U‐shaped neck cushion for 1 h per day until the day before surgery.

6. Patients perform orofacial training: alternating between blowing, laughing, and grinning exercises (15 min, three times a day).

In this study, rigorous monitoring of patient compliance with the MTTE protocol was essential for ensuring adherence and evaluating its effect on postoperative outcomes, particularly dysphagia. Patients received comprehensive preoperative education, including detailed instructions and instructional materials to reinforce correct practice. Compliance was tracked through daily logs, which were regularly reviewed during preoperative visits and postoperative follow‐up appointments. Regular check‐ins, both preoperative and postoperative, helped address any issues and reinforce the importance of adherence. Positive reinforcement and goal setting were used to motivate patients. High compliance with the MTTE protocol might be associated with a significant reduction in postoperative dysphagia incidence, faster recovery times, and lower rates of complications such as choking, cough, and hoarseness. The methods used to ensure adherence not only validated the study's findings but also empowered patients to take an active role in their recovery, enhancing their overall healthcare experience.

### Clinical Assessment

Demographic data including sex, age, body mass index (BMI), hypertension, diabetes, smoking, and preoperative hospitalization were collected. Blood loss, duration of surgery, number of instrumented segments, functional outcome swallowing scale (FOSS) scores, perioperative complications (hoarseness, choke or cough when drinking water, surgical site infections, and cerebrospinal fluid leakage), and postoperative hospitalization were recorded. The cervical Japanese Orthopedic Association (c‐JOA) score was used to evaluate neurological function, preoperatively, postoperatively, and during the 6‐month follow‐up.

### Functional Outcome Swallowing Scale

Dysphagia severity was assessed *via* a telephone evaluation, quantitatively, according to FOSS. FOSS is a functional outcome swallowing scale that is grouped into six categories (stage 0 for normal, stage 1 for episodic dysphagia, stage 2 for dysphagia with nobody weight (BWI) change, Stage 3 for body weight loss <10%, stage 4 for body weight loss >10%, and stage 5 for non‐oral feeding).^19^

### Control of Intraoperative Factors

In this study, several intraoperative factors, such as surgeon experience and specific surgical techniques, were carefully controlled to minimize their effect on postoperative outcomes, particularly dysphagia. All surgeries were performed by a team of highly experienced surgeons, each with a minimum of 10 years of experience and at least 200 prior ACDF surgeries. Operators underwent specific training sessions to ensure uniformity in the execution of both TTTE and MTTE, with continuous monitoring and periodic audits to ensure adherence to standardized protocols. Detailed preoperative planning was conducted for each patient, and surgical steps for both TTTE and MTTE were meticulously standardized, including the placement and duration of retraction, and achieving cervical hyperextension. Consistent intraoperative monitoring tools and uniform anesthesia protocols were used across all cases to ensure reliable data collection and patient response. Postoperative care was standardized, including pain management and follow‐up assessments using the FOSS and c‐JOA scores, with close monitoring of patient compliance to postoperative instructions. Data were collected using standardized forms and protocols, and outcome assessors were blinded to the type of tracheal traction exercise performed to reduce bias.

### Cervical‐Japanese Orthopedic Association

The clinical condition of patients was assessed using the C‐JOA (the highest possible score is 17 points).^21^ The C‐JOA comprises four major aspects: the motor‐function of the upper limbs; the motor‐function of the lower limbs; the sensory function of the upper limbs, lower limbs, and trunk; and bladder function. For details of the cervical‐JOA, see reference.^20^

### Statistical Analysis

Data analysis was conducted using SPSS software (Version 26.0, IBM, Armonk, NY, USA). Continuous variables that followed a normal distribution were reported as mean ± standard deviation, while those not normally distributed were presented as median (interquartile range). Statistical comparisons of continuous variables between two groups were performed using either the independent samples *t*‐test or the Mann–Whitney U‐test, depending on the distribution. Categorical data from two groups were analyzed with the *χ* ^2^‐test, and ordinal data were evaluated using the Wilcoxon rank‐sum test. A significance level of *α* = 0.05 was set, with *p*‐values <0.05 deemed indicative of statistical significance.

## Results

### Demographic Data

A total of 143 patients participated in this research and multilevel fusions were carried out for all patients. The demographic characteristics were balanced in the group MTTE and the group TTTE. The numbers of patients were 75 and 68 in group MTTE and group TTTE, respectively, including 40 men and 35 women in group MTTE with an average age of 51.43 ± 11.25 years and 32 men and 36 women in group TTTE with an average age of 52.35 ± 10.43 years. No significant differences in BMI, hypertension, diabetes, and smoking status were observed between the group MTTE and the group TTTE (*p* > 0.05). No significant differences were observed in the number of fusion segments and the preoperative hospitalization days between the group MTTE and group TTTE (*p* > 0.05). The mean surgical duration was 159.04 ± 9.82 and 164.41 ± 12.22 min in group MTTE and group TTTE, respectively (*p* < 0.05) (Table 1).

**TABLE 1:** Demographic characteristics of patients.

Variables	Group MTTE (*n* = 75)	Group TTTE (*n* = 68)	*χ* ^2^/*Z*	*p*‐value
Age, mean ± SD, year	51.43 ± 11.25	52.35 ± 10.43	1.817	0.611
Gender, no. (%)				
Male	40 (53.33)	32 (47.06)	0.339	0.454
Female	35 (46.67)	36 (52.94)		
BMI, mean ± SD	25.29 ± 3.63	24.19 ± 3.66	1.841	0.074
Diabetes, no. (%)	22 (29.33)	19 (27.94)	0	0.854
Hypertension, no. (%)	24 (32.00)	22 (32.35)	0	0.964
Smoker, no. (%)	28 (37.33)	23 (33.82)	0.069	0.662
Number of fusion segments				
3 segments	62 (82.67)	58 (85.29)	0.04	0.669
4 segments	13 (17.33)	10 (14.71)		
Preoperative hospitalization, day	3.11 ± 0.45	3.15 ± 0.55	2.782	0.632

Abbreviation: BMI, body mass index.

### Clinical Outcomes

The clinical outcomes were presented in Table 2. Group MTTE exhibited significantly lower incidence of dysphagia than group TTTE (25.33%, 44.12%; *p* = 0.018). In addition, significant differences were observed in blood loss (102.03 ± 17.04, 113.46 ± 14.92; *p* < 0.001) and surgical duration (159.04 ± 9.82, 164.41 ± 12.22; *p* = 0.004) between the MTTE group and the TTTE group. Further, significant differences were observed in postoperative complications such as choking cough (*p* = 0.020), cerebrospinal fluid leakage (*p* = 0.010), and hoarseness (*p* = 0.006) between the two groups, but there was no obvious difference in surgical site infections (*p* > 0.05) between the two groups. There were no significant differences in c‐JOA score between the two groups before surgery (*p* > 0.05) (Table 2). No significant difference was observed in the preoperative c‐JOA score between the two groups (*p* > 0.05). The c‐JOA scores at 2 weeks (*p* = 0.005) and 6 weeks (*p* = 0.016) after the operations of the MTTE group were significantly higher than those in the TTTE group at different time points after surgery. However, no significant differences were observed in the c‐JOA scores at 12 and 24 weeks after surgery (*p* > 0.05) between the two groups.

**TABLE 2:** Surgery‐related information.

Variables	Group MTTE (*n* = 75)	Group TTTE (*n* = 68)	*χ* ^2^/*Z*	*p*‐value
Dysphagia, no (%)	19 (25.33)	30 (44.12)	4.784	0.018
Blood loss, mL	102.03 ± 17.04	113.46 ± 14.92	15.709	0.000
Surgical duration, min	159.04 ± 9.82	164.41 ± 12.22	1.016	0.004
Other complications, no (%)				
Surgical site infections	1 (1.33)	2 (2.94)	0.449	0.503
Choking cough	4 (5.33)	12 (17.65)	5.442	0.020
Cerebrospinal fluid leakage	2 (2.67)	10 (14.71)	6.724	0.010
Hoarseness	4 (5.33)	14 (20.59)	7.543	0.006
C‐JOA scores				
Preoperative	6.55 ± 2.46	7.12 ± 2.84	2.528	0.200
2 weeks	9.29 ± 1.52	8.54 ± 1.65	0.083	0.005
6 weeks	11.03 ± 2.12	10.21 ± 1.87	1.556	0.016
12 weeks	12.56 ± 1.65	12.18 ± 1.53	1.232	0.152
24 weeks	14.05 ± 0.97	13.72 ± 1.33	10.719	0.087

Abbreviation: C‐JOA scores, cervical Japanese Orthopedic Association score.

### The Severity of Dysphagia

**FIGURE 2:**
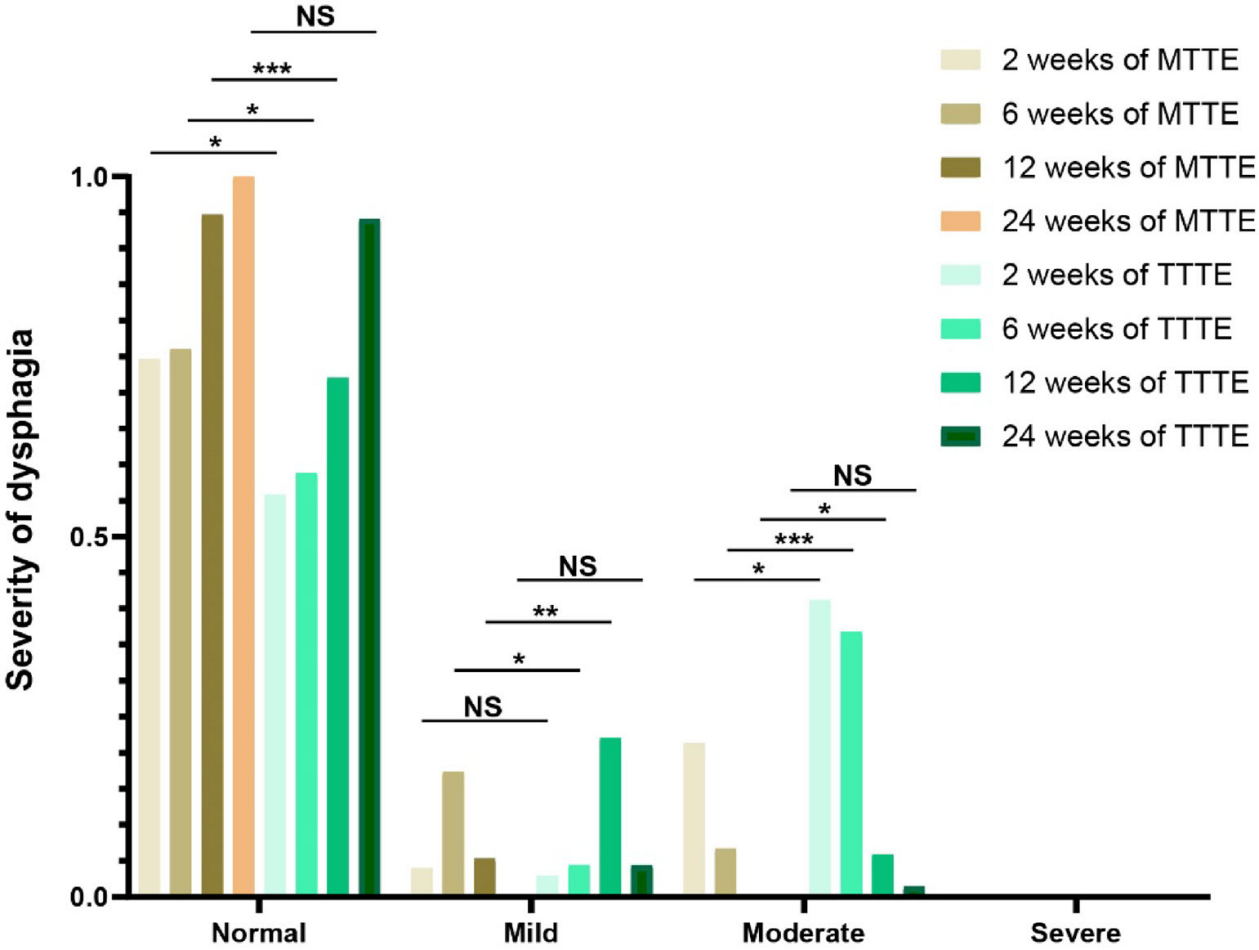
The severity of dysphagia in patients.

Severity of dysphagia was evaluated by FOSS. According to the FOSS score, patients were marked with different severity of dysphagia, including normal (FOSS = 0), mild (FOSS = 1), moderate (FOSS = 2 or 3), or severe (FOSS = 4 or 5), and the results of this study are presented in the Figure 2. The results showed that the MTTE group exhibited a significantly higher percentage of “normal” in FOSS scores at 2 weeks (*p* < 0.05), 6 weeks (*p* < 0.05), and 12 weeks (*p* < 0.001) than the TTTE group. The MTTE group had a remarkably higher percentage of “mild dysphagia” at 6 weeks after surgery but a significantly lower percentage of “mild dysphagia” at 12 weeks than the TTTE group. Importantly, significant differences in percentage of “moderate dysphagia” at 2 weeks (*p* < 0.05), 6 weeks (*p* < 0.001), and 12 weeks (*p* < 0.05) were observed in the MTTE group when compared with the TTTE group. No case of severe dysphagia was observed in either the MTTE group or the TTTE group.

### Factors Contributing to the Dysphagia in this Study

To further explore the factors associated with the incidence of dysphagia in patients with multilevel ACDF, univariate logistic regression and multivariate logistic regression analysis were performed. The results of both univariate logistic regression analysis showed that MTTE, age, and BMI were significantly relevant to the incidence of dysphagia (Table 3). In addition, multivariate logistic regression analysis revealed that MTTE, age, and BMI were significantly associated with dysphagia in patients receiving multilevel ACDF (Table 4).

**Table 3:** Univariate logistic regression analysis.

Variables	Dysphagia (*n* = 49)	No dysphagia (*n* = 94)	*χ* ^2^/*Z*	*p*‐value
MTTE/TTTE				
MTTE	19	56		
TTTE	30	38	−2.341	0.019
Sex				
Male	26	46		
Female	23	48	−0.468	0.640
Age, year				
≤40	5	27		
>40 and ≤60	32	51	2.274	0.023
>60	12	16	2.260	0.024
BMI				
<24	19	49		
≥24 and <28	15	29	0.690	0.490
≥28	15	16	1.963	0.049
Hypertension				
No	34	63		
Yes	15	31	−0.287	0.774
Diabetes				
No	38	64		
Yes	11	30	−1.182	0.237
Smoking				
No	29	63		
Yes	20	31	0.927	0.354
Surgical segments				
3	40	80		
4	9	14	0.536	0.592
Preoperative hospitalization				
≤2	6	21		
3	31	51	1.463	0.143
>3	12	22	1.104	0.270
Blood loss				
≤100	11	35		
>100, ≤120	26	40	1.699	0.089
>120	12	19	1.381	0.167
Surgical duration				
≤160	19	43		
>160	30	51	0.797	0.425

**Table 4:** Multivariate logistic regression analysis

Intercept and variables	*β*	OR (95% CI)	*p*‐value
Intercept	−2.642	0.071 (0.010, 0.441)	0.006
MTTE_TTTE			
MTTE			
TTTE	−0.854	0.426 (0.181, 0.969)	0.044
Sex			
Male			
Female	−0.001	0.999 (0.442, 2.262)	0.997
Age, years			
≤40			
>40 and ≤60	1.205	3.338 (1.129,11.640)	0.040
>60	1.250	3.489 (0.956, 14.342)	0.067
BMI			
<4			
≥24 and <28	0.458	1.581 (0.642, 3.916)	0.318
≥28	1.378	3.968 (1.445, 11.530)	0.009
Hypertension			
No			
Yes	−0.141	0.869 (0.358, 2.063)	0.751
Diabetes			
No			
Yes	−0.600	0.549 (0.199, 1.417)	0.226
Smoking			
No			
Yes	0.226	1.254 (0.549, 2.863)	0.589
Surgical segments			
3			
4	0.042	1.043 (0.360, 2.920)	0.937
Preoperative hospitalization, day			
≤2			
3	0.609	1.839 (0.587, 6.381)	0.310
>3	0.482	1.620 (0.442, 6.372)	0.473
Blood loss			
≤100			
>100, ≤120	0.749	2.115 (0.837, 5.618)	0.120
>120	0.332	1.394 (0.418, 4.631)	0.585
Surgical duration			
≤160			
>160	0.255	1.291 (0.578, 2.923)	0.534

*Note*: *β* is the regression coefficient.

Abbreviations: CI, confidence interval; OR, odds ratio.

## Discussion

### Main Findings

The primary aim of this study was to compare the outcomes of MTTE and TTTE in patients undergoing multilevel ACDF. Major results revealed balanced demographic characteristics between MTTE and TTTE groups. Notably, MTTE demonstrated superior clinical outcomes, including significantly lower dysphagia incidence, reduced blood loss, shorter surgical durations, and varied postoperative complications compared to TTTE. These findings hold significant clinical implications in optimizing surgical approaches and improving patient outcomes in multilevel ACDF procedures.

### Comparison with Prior Dysphagia Mitigation Techniques

Previous studies reported that many methods might decrease the incidence of postoperative dysphagia, which include steroid administration and lowering the endotracheal tube cuff pressure. Curto *et al*. reported that the administration of steroids can not only reduce the incidence of dysphagia but also meliorate the severity of dysphagia after cervical disk replacement surgery.^12^ The endotracheal tube cuff pressure is associated with dysphagia after ACDF. A prospective study reported that compared with the control group, an endotracheal tube cuff pressure at 20 mmHg can significantly decrease the incidence of dysphagia, hoarseness, and dysphonia.^13^ However, these measures cannot reduce the incidence of DAACS to a satisfactory level. In 2012, Chen *et al*. reported that the tracheal traction exercise played a pivotal role in preventing postoperative dysphagia.^10^ Their results indicated that tracheal traction exercise can help decrease the incidence of postoperative dysphagia through improving the compliance of the trachea and esophagus. Although their study included patients undergoing both single‐level and multilevel cervical spine surgery, the number of patients in each group is limited. In addition, the postoperative investigation of patients was first conducted 6 weeks after surgery in their study. These limitations might lead to inaccurate conclusions.

Although TTTE has been effective for many patients, those undergoing multilevel fusion often struggle with adaptation to postoperative conditions, particularly due to significant increases in cervical lordosis and the overuse of metal retractors in the tracheoesophageal groove. These factors can impair swallowing by causing excessive cervical curvature and damage to the esophagus. To address the limitations of TTTE and mitigate the risks of DAACS after multilevel ACDF, MTTE was developed. MTTE introduces several improvements: chewing sugar‐free gum to stimulate saliva and enhance swallowing reflexes, using a U‐shaped neck cushion to achieve cervical hyperextension and minimize tracheal and esophageal tension, and incorporating orofacial exercises to strengthen the muscles involved in swallowing. These adaptations aim to improve patient recovery by promoting better oral hygiene, optimizing surgical alignment, and enhancing neuromuscular coordination. MTTE offers a comprehensive approach that not only mitigates the risks of TTTE but also facilitates a smoother and more effective postoperative recovery for patients undergoing multilevel ACDF.

Cervical curvature tends to increase following the ACDF procedure. There are several reasons for this. First, in clinical practice, to obtain a better operative field of vision and maintain cervical spine stabilization, the patient tends to be placed in supine position with neck extension by placing a cushion under the shoulder. This might lead to increased cervical lordosis. Second, it is widely recognized that cervical lordosis is the biomechanically ideal alignment, and the loss of cervical lordosis can lead to disrupted biomechanics. In clinical practice, to maintain the postoperative cervical lordosis, surgeons might deliberately maintain the lordotic curvature using a neck extension position in the procedure. However, this is often dependent on the experience of the surgeons, which might be influenced by various factors. In our study, we focused on patients undergoing three or four levels of ACDF and conducted a larger‐scale investigation starting from 2 weeks post‐surgery.

### Modified Tracheal Traction Exercise Helps Reduce the Incidence of Postoperative Dysphagia

In anterior cervical surgical operations, maintaining a clear view and access to the operative field is crucial. Adequate intraoperative retraction of the trachea and esophagus is necessary to provide sufficient space for the procedure. However, increased retractor pressure can lead to postoperative dysphagia due to maladaptation of intraoperative traction.^10^ The maladaptation might result in hyperemia, oedema, hemorrhage or ischemia of the trachea, esophagus or the surrounding soft tissues, which consequently increased the incidence rate of dysphagia. It was reported that, compared with the incidence of dysphagia in patients without traction of the trachea and esophagus before anterior approach, the rate in patients with pre‐tractions was considerably reduced.^10^ However, even with pre‐traction, some patients, especially those undergoing multilevel fusion, still experienced postoperative dysphagia.^10^ In our study, we modified TTTE to further reduce the incidence of dysphagia by introducing MTTE.

In comparing MTTE to TTTE in multilevel ACDF, MTTE resulted in significantly lower blood loss (102.03 ± 17.04 mL vs. 113.46 ± 14.92 mL, *p* < 0.001) and shorter surgical duration (159.04 ± 9.82 min vs. 164.41 ± 12.22 min, *p* = 0.004). MTTE improved compliance of the trachea and esophagus, leading to a smoother and safer procedure. Complications like choking, cough, and hoarseness were less frequent in the MTTE group, with hoarseness notably lower (5.33% vs. 20.59%, *p* = 0.006), suggesting less risk of injury to the superior laryngeal and recurrent laryngeal nerves. These findings indicate that MTTE is more effective than TTTE in minimizing postoperative dysphagia and complications, enhancing surgical safety and efficiency for ACDF patients. The traditional method required more force to maintain a good operative field, increasing the risk of damaging the esophagus, nerves, and blood vessels. The findings in this present study demonstrated that MTTE, by softening the prevertebral soft tissue and esophagus, might help reduce the resistance against intraoperative traction. MTTE led to decreased overtraction on these tissues, shortening the operative time and reducing the risk of injuries to the prevertebral soft tissue, esophagus, recurrent laryngeal nerve, and superior laryngeal nerve. Specifically, the incidence of hoarseness was significantly lower in the MTTE group (5.33%) compared to the TTTE group (20.59%, *p* = 0.006). In addition, it was reported that cervical hyperextension in anterior cervical surgery might cause dysphagia.^21^ The dC2–C7 angle is significantly related to early DAACS, and the rate of DAACS was increased when the dC2–C7 angle was >9°.^9^ The over‐increase in cervical lordosis might result in protruding of the posterior pharyngeal wall; and this could reduce pharyngeal space and affect the pharyngeal squeeze and laryngeal elevation.^4^ The over‐increase in the cervical sagittal alignment might increase the incidence of dysphagia. The MTTE approach, which included maintaining slight hyperextension and promoting swallowing during the exercise, helped patients adapt to the postoperative state, further reducing dysphagia incidence.

### Limitations and Strengths of the Study

The limitations of our study must be acknowledged. First, the retrospective single‐center design might limit the generalizability of findings to broader populations or other healthcare settings. Besides, although rigorous strategies have been carried out, recall bias in assessing dysphagia severity *via* telephone evaluation may have been present in this study and objective assessment methods in future studies are required to mitigate recall bias. Additionally, unmeasured confounders, such as individual anatomical variations or surgeon expertise, might influence outcomes. Furthermore, the relatively short follow‐up duration restricted the assessment of long‐term outcomes and potential late complications, necessitating extended monitoring for comprehensive evaluation.

Despite these limitations, our study also has several strengths. The focus on patients undergoing three or four levels of ACDF enhances the reliability of our findings. The early postoperative investigation starting from 2 weeks after surgery allowed for the timely detection of dysphagia and other complications. The implementation of MTTE with a standardized protocol contributed to a more consistent assessment of its effectiveness compared to TTTE. These strengths bolster the validity of our results and provide a solid foundation for future research in optimizing surgical techniques for ACDF.

## Conclusion

In conclusion, this study underscores the favorable outcomes of MTTE over TTTE in multilevel ACDF, particularly in reducing dysphagia incidence, blood loss, and specific postoperative complications. While consistent with prior evidence, disparities emphasize the need for standardized methodologies and collaborative multi‐center studies with larger cohorts and extended follow‐ups to validate and elucidate these findings further. Addressing these disparities will refine our understanding and enhance the applicability of optimal surgical techniques in multilevel ACDF procedures.

## Conflict of Interest Statement

No competing financial interests or personal relationships need to be reported.

## Ethics Statement

The ethics committee approval was obtained from Ethics Committee of Shanghai Changzheng Hospital.

## Author Contributions

JSW, KQS and JGS are responsible for the conception of the study. JSW, FDL, and KQS led protocol development, supported by all authors. JSW, FDL, and JCS lead on data analyses. All authors have contributed to data interpretation, conclusions, and dissemination. JSW and FDL drafted the initial manuscript. Subsequent drafts were developed with ZJZ, RS, and KQS. All authors have read, contributed to, and agreed upon the final manuscript.

## Data Availability

The raw data can be provided by the corresponding author upon reasonable request.
